# Are energy and protein targets being met in the ICU?

**DOI:** 10.1186/2197-425X-3-S1-A578

**Published:** 2015-10-01

**Authors:** R Booth, L Morgan

**Affiliations:** Royal Gwent Hospital, Dietetics, Newport, United Kingdom

## Background

Multiple barriers exist to achieving energy and protein targets in the critically ill. Elke et al [1] used data from ICU's worldwide which estimated average energy delivery of only 1057 kcals/day and protein delivery 49 g/d (0.7 g/kg/d). Weijs et al [2] demonstrated that meeting energy and protein requirements is associated with decreased mortality. Local data from 2012 found an average delivery of 1424 kCal and 56.8 g protein/d (0.75 g/kg/d) once at target rate (TR) and that 40% of patients were taking > 5 days to reach TR. As a result alterations were made to the ICU feeding protocol; 1.GRV threshold increased to 300 ml, 2. Daily monitoring of energy and protein provision, 3.Introduction of protein modules.

## Objectives

To assess energy and protein delivery for enterally fed patients in a 16 bed ICU following implementation of modifications to protocol, and comparison with historical controls.

## Methods

A retrospective, case control study over 2 weeks in the ICU. Energy and protein intakes recorded from admission up until day 20; or until discharge from critical care, removal of the enteral feeding tube or death.

## Results

20 patients, 11 male + 9 female, aged 23-84 yrs. Mean weight 80 kg + BMI 27.8 kg/m^2^ (19-41 kg/m^2^). 79% of patients had EN initiated within 48 hrs and 95% within 72 hrs. Mean time to TR was 4 days. 19% of patients took >5 days to reach TR. Mean daily energy delivery 1428 kCal + 93% energy requirements met. Mean daily protein delivery 64.7 g (0.86 g/kg/d) + 71%of protein requirements met.

## Conclusions

Increasing GRV threshold improved the number of patients reaching TR in 5 days by 50%... Energy delivery was similar to 2012 but higher than those reported by Elke et al [1]. However % of energy requirements met once at TR increased by 4% from 2012. Protein delivery increased by 8 g protein /d (13%) and was 15.7 g (22%) higher than stated by Elke et al. The use of protein modules and daily monitoring improves energy and protein delivery. However, protein is still falling short of the recommended 1.5 g/kg/day [3] due to non-feed sources of energy and high requirements in the obese; ≥ 2-2.5 g/kg IBW (ASPEN, 2009).
